# Supplementary material for: The response of three-dimensional pancreatic alpha and beta cell co-cultures to oxidative stress
Source: PLoS One. 2022 Mar 15;17(3):e0257578. doi: 10.1371/journal.pone.0257578 (PMC8923503; doi:10.1371/journal.pone.0257578)
Supplement: S1 Table — (DOCX) [file pone.0257578.s001.docx]

**Table S1**: **Statistical significance (t-test) of cell viability in monolayers upon induction of oxidative stress by H_2_O_2_ (20–2000 μM) compared to the control (0 μM H_2_O_2_) (from Fig 1A).**

|  | **Ratio INS1E:alphaTC1** | | | | |
| --- | --- | --- | --- | --- | --- |
| **[H_2_O_2_] (μM)** | **0:100** | **20:80** | **50:50** | **80:20** | **100:0** |
| 20 | 0.203 | 0.168 | 0.691 | 0.284 | 0.725 |
| 100 | <0.001 | 0.085 | 0.061 | 0.238 | 0.177 |
| 500 | <0.001 | <0.001 | <0.001 | 0.339 | 0.438 |
| 1000 | <0.001 | <0.001 | <0.001 | <0.001 | <0.001 |
| 2000 | <0.001 | <0.001 | <0.001 | <0.001 | <0.001 |
