# Supplementary material for: The response of three-dimensional pancreatic alpha and beta cell co-cultures to oxidative stress
Source: PLoS One. 2022 Mar 15;17(3):e0257578. doi: 10.1371/journal.pone.0257578 (PMC8923503; doi:10.1371/journal.pone.0257578)
Supplement: S2 Table — (DOCX) [file pone.0257578.s002.docx]

**Table S2**: **Statistical significance (t-test) of cell viability in 3D aggregates upon induction of oxidative stress by H_2_O_2_ (20–2000 μM) compared to the control (0 μM H_2_O_2_) (from Fig 1B).**

|  | **Ratio INS1E:alphaTC1** | | | | |
| --- | --- | --- | --- | --- | --- |
| **[H_2_O_2_] (μM)** | **0:100** | **20:80** | **50:50** | **80:20** | **100:0** |
| 20 | 0.893 | 0.500 | 0.822 | 0.496 | 0.064 |
| 100 | 0.001 | 0.958 | 0.582 | 0.800 | 0.305 |
| 500 | <0.001 | <0.001 | 0.946 | 0.215 | 0.672 |
| 1000 | <0.001 | <0.001 | <0.001 | <0.001 | <0.001 |
| 2000 | <0.001 | <0.001 | <0.001 | <0.001 | <0.001 |
