# Supplementary material for: The response of three-dimensional pancreatic alpha and beta cell co-cultures to oxidative stress
Source: PLoS One. 2022 Mar 15;17(3):e0257578. doi: 10.1371/journal.pone.0257578 (PMC8923503; doi:10.1371/journal.pone.0257578)
Supplement: S3 Table — (DOCX) [file pone.0257578.s003.docx]

**Table S3. Statistical significance (t-test) of oxidative stress in monolayers upon induction by H_2_O_2_ (20–2000 μM) compared to the control (0 μM H_2_O_2_).**

|  | **Ratio INS1E:alphaTC1** | | | | |
| --- | --- | --- | --- | --- | --- |
| **[H_2_O_2_] (μM)** | **0:100** | **20:80** | **50:50** | **80:20** | **100:0** |
| 20 | 0.836 | 0.913 | 0.612 | 0.309 | 0.751 |
| 100 | 0.919 | 0.623 | 0.961 | 0.078 | 0.967 |
| 500 | 0.981 | 0.042 | 0.477 | 0.080 | 0.996 |
| 1000 | 0.656 | 0.006 | 0.201 | <0.001 | 0.324 |
| 2000 | 0.595 | 0.012 | 0.221 | <0.001 | 0.540 |
