# Supplementary material for: The response of three-dimensional pancreatic alpha and beta cell co-cultures to oxidative stress
Source: PLoS One. 2022 Mar 15;17(3):e0257578. doi: 10.1371/journal.pone.0257578 (PMC8923503; doi:10.1371/journal.pone.0257578)
Supplement: S4 Table — (DOCX) [file pone.0257578.s004.docx]

**Table S4. Statistical significance (t-test) of oxidative stress in 3D aggregates upon induction by H_2_O_2_ (20–2000 μM) compared to the control (0 μM H_2_O_2_).**

|  | **Ratio INS1E:alphaTC1** | | | | |
| --- | --- | --- | --- | --- | --- |
| **[H_2_O_2_] (μM)** | **0:100** | **20:80** | **50:50** | **80:20** | **100:0** |
| 20 | 0.888 | 0.569 | 0.721 | 0.562 | 0.564 |
| 100 | 0.259 | 0.605 | 0.511 | 0.758 | 0.994 |
| 500 | 0.555 | 0.653 | 0.483 | 0.582 | 0.975 |
| 1000 | 0.516 | 0.624 | 0.290 | 0.683 | 0.379 |
| 2000 | 0.804 | 0.412 | 0.178 | 0.998 | 0.104 |
