# Supplementary material for: The response of three-dimensional pancreatic alpha and beta cell co-cultures to oxidative stress
Source: PLoS One. 2022 Mar 15;17(3):e0257578. doi: 10.1371/journal.pone.0257578 (PMC8923503; doi:10.1371/journal.pone.0257578)
Supplement: S5 Table — (DOCX) [file pone.0257578.s005.docx]

**Table S5. Statistical significance (t-test) of the oxidative stress positive INS1E cells in monolayer co-cultures when exposed to 0–2000 μM H_2_O_2_.**

|  |  | **Ratio INS1E:alphaTC1** | | | | |
| --- | --- | --- | --- | --- | --- | --- |
|  |  | **0:100** | **20:80** | **50:50** | **80:20** | **100:0** |
| 0 μM | **0:100** | -- | <0.001 | <0.001 | <0.001 | <0.001 |
|  | **20:80** | -- | -- | 0.045 | 0.045 | 0.864 |
|  | **50:50** | -- | -- | -- | 0.005 | 0.199 |
|  | **80:20** | -- | -- | -- | -- | 0.295 |
|  | **100:0** | -- | -- | -- | -- | -- |
|  | | | | | | |
|  |  | **0:100** | **20:80** | **50:50** | **80:20** | **100:0** |
| 20 μM | **0:100** | -- | <0.001 | <0.001 | <0.001 | <0.001 |
|  | **20:80** | -- | -- | 0.144 | 0.385 | 0.734 |
|  | **50:50** | -- | -- | -- | 0.054 | 0.184 |
|  | **80:20** | -- | -- | -- | -- | 0.738 |
|  | **100:0** | -- | -- | -- | -- | -- |
|  | | | | | | |
|  |  | **0:100** | **20:80** | **50:50** | **80:20** | **100:0** |
| 100 μM | **0:100** | -- | <0.001 | <0.001 | <0.001 | <0.001 |
|  | **20:80** | -- | -- | 0.178 | 0.547 | 0.459 |
|  | **50:50** | -- | -- | -- | 0.614 | 0.106 |
|  | **80:20** | -- | -- | -- | -- | 0.350 |
|  | **100:0** | -- | -- | -- | -- | -- |
|  | | | | | | |
|  |  | **0:100** | **20:80** | **50:50** | **80:20** | **100:0** |
| 500 μM | **0:100** | -- | <0.001 | <0.001 | <0.001 | <0.001 |
|  | **20:80** | -- | -- | 0.701 | 0.114 | 0.147 |
|  | **50:50** | -- | -- | -- | 0.424 | 0.359 |
|  | **80:20** | -- | -- | -- | -- | 0.359 |
|  | **100:0** | -- | -- | -- | -- | -- |
|  | | | | | | |
|  |  | **0:100** | **20:80** | **50:50** | **80:20** | **100:0** |
| 1000 μM | **0:100** | -- | <0.001 | <0.001 | <0.001 | <0.001 |
|  | **20:80** | -- | -- | 0.936 | <0.001 | <0.001 |
|  | **50:50** | -- | -- | -- | 0.030 | 0.001 |
|  | **80:20** | -- | -- | -- | -- | <0.001 |
|  | **100:0** | -- | -- | -- | -- | -- |
|  | | | | | | |
|  |  | **0:100** | **20:80** | **50:50** | **80:20** | **100:0** |
| 2000 μM | **0:100** | -- | <0.001 | <0.001 | <0.001 | <0.001 |
|  | **20:80** | -- | -- | 0.714 | 0.002 | <0.001 |
|  | **50:50** | -- | -- | -- | 0.103 | 0.005 |
|  | **80:20** | -- | -- | -- | -- | <0.001 |
|  | **100:0** | -- | -- | -- | -- | -- |
