# Supplementary material for: The response of three-dimensional pancreatic alpha and beta cell co-cultures to oxidative stress
Source: PLoS One. 2022 Mar 15;17(3):e0257578. doi: 10.1371/journal.pone.0257578 (PMC8923503; doi:10.1371/journal.pone.0257578)
Supplement: S6 Table — (DOCX) [file pone.0257578.s006.docx]

**Table S6. Statistical significance (t-test) of the oxidative stress positive alphaTC1 cells in monolayer co-cultures when exposed to 0–2000 μM H_2_O_2_.**

|  |  | **Ratio INS1E:alphaTC1** | | | | |
| --- | --- | --- | --- | --- | --- | --- |
|  |  | **0:100** | **20:80** | **50:50** | **80:20** | **100:0** |
| 0 μM | **0:100** | -- | 0.217 | 0.012 | <0.001 | <0.001 |
|  | **20:80** | -- | -- | <0.001 | <0.001 | <0.001 |
|  | **50:50** | -- | -- | -- | 0.097 | <0.001 |
|  | **80:20** | -- | -- | -- | -- | <0.001 |
|  | **100:0** | -- | -- | -- | -- | -- |
|  | | | | | | |
|  |  | **0:100** | **20:80** | **50:50** | **80:20** | **100:0** |
| 20 μM | **0:100** | -- | 0.264 | 0.115 | <0.001 | <0.001 |
|  | **20:80** | -- | -- | 0.006 | <0.001 | <0.001 |
|  | **50:50** | -- | -- | -- | 0.009 | <0.001 |
|  | **80:20** | -- | -- | -- | -- | <0.001 |
|  | **100:0** | -- | -- | -- | -- | -- |
|  | | | | | | |
|  |  | **0:100** | **20:80** | **50:50** | **80:20** | **100:0** |
| 100 μM | **0:100** | -- | 0.174 | 0.037 | 0.015 | <0.001 |
|  | **20:80** | -- | -- | <0.001 | <0.001 | <0.001 |
|  | **50:50** | -- | -- | -- | 0.408 | <0.001 |
|  | **80:20** | -- | -- | -- | -- | <0.001 |
|  | **100:0** | -- | -- | -- | -- | -- |
|  | | | | | | |
|  |  | **0:100** | **20:80** | **50:50** | **80:20** | **100:0** |
| 500 μM | **0:100** | -- | 0.005 | 0.163 | 0.009 | <0.001 |
|  | **20:80** | -- | -- | <0.001 | <0.001 | <0.001 |
|  | **50:50** | -- | -- | -- | 0.029 | <0.001 |
|  | **80:20** | -- | -- | -- | -- | <0.001 |
|  | **100:0** | -- | -- | -- | -- | -- |
|  | | | | | | |
|  |  | **0:100** | **20:80** | **50:50** | **80:20** | **100:0** |
| 1000 μM | **0:100** | -- | 0.002 | 0.477 | 0.852 | <0.001 |
|  | **20:80** | -- | -- | <0.001 | 0.002 | <0.001 |
|  | **50:50** | -- | -- | -- | 0.367 | <0.001 |
|  | **80:20** | -- | -- | -- | -- | <0.001 |
|  | **100:0** | -- | -- | -- | -- | -- |
|  | | | | | | |
|  |  | **0:100** | **20:80** | **50:50** | **80:20** | **100:0** |
| 2000 μM | **0:100** | -- | 0.003 | 0.408 | 0.473 | <0.001 |
|  | **20:80** | -- | -- | 0.001 | 0.014 | <0.001 |
|  | **50:50** | -- | -- | -- | 0.134 | <0.001 |
|  | **80:20** | -- | -- | -- | -- | <0.001 |
|  | **100:0** | -- | -- | -- | -- | -- |
