# Supplementary material for: The response of three-dimensional pancreatic alpha and beta cell co-cultures to oxidative stress
Source: PLoS One. 2022 Mar 15;17(3):e0257578. doi: 10.1371/journal.pone.0257578 (PMC8923503; doi:10.1371/journal.pone.0257578)
Supplement: S7 Table — (DOCX) [file pone.0257578.s007.docx]

**Table S7. Statistical significance (t-test) of the intracellular GSH levels in monolayer co-cultures when exposed to 500 μM H_2_O_2_ (Fig 3).**

|  |  | **0 μM H_2_O_2_** | | | **500 μM H_2_O_2_** | | |
| --- | --- | --- | --- | --- | --- | --- | --- |
|  | **INS1E:alphaTC1** | **0:100** | **50:50** | **100:0** | **0:100** | **50:50** | **100:0** |
| **0 μM H_2_O_2_** | **0:100** | -- | 0.203 | 0.611 | 0.005 | -- | -- |
|  | **50:50** | -- | -- | 0.240 | -- | 0.022 | -- |
|  | **100:0** | -- | -- | -- | -- | -- | 0.392 |
| **500 μM H_2_O_2_** | **0:100** | -- | -- | -- | -- | 0.832 | 0.807 |
|  | **50:50** | -- | -- | -- | -- | -- | 0.592 |
|  | **100:0** | -- | -- | -- | -- | -- | -- |
| **0 μM H_2_O_2_** | **0:100** | -- | 0.510 | 0.321 | 0.279 | -- | -- |
|  | **50:50** | -- | -- | 0.330 | -- | 0.181 | -- |
|  | **100:0** | -- | -- | -- | -- | -- | 0.655 |
| **500 μM H_2_O_2_** | **0:100** | -- | -- | -- | -- | 0.726 | 0.868 |
|  | **50:50** | -- | -- | -- | -- | -- | 0.861 |
|  | **100:0** | -- | -- | -- | -- | -- | -- |
