# Supplementary material for: The response of three-dimensional pancreatic alpha and beta cell co-cultures to oxidative stress
Source: PLoS One. 2022 Mar 15;17(3):e0257578. doi: 10.1371/journal.pone.0257578 (PMC8923503; doi:10.1371/journal.pone.0257578)
Supplement: S8 Table — (DOCX) [file pone.0257578.s008.docx]

**Table S8. Statistical significance (t-test) of the oxidative stress positive INS1E cells in 3D aggregate co-cultures when exposed to 0–2000 μM H_2_O_2_.**

|  |  | **Ratio INS1E:alphaTC1** | | | | |
| --- | --- | --- | --- | --- | --- | --- |
|  |  | **0:100** | **20:80** | **50:50** | **80:20** | **100:0** |
| 0 μM | **0:100** | -- | <0.001 | 0.009 | <0.001 | <0.001 |
|  | **20:80** | -- | -- | 0.023 | 0.234 | 0.037 |
|  | **50:50** | -- | -- | -- | <0.001 | 0.211 |
|  | **80:20** | -- | -- | -- | -- | <0.001 |
|  | **100:0** | -- | -- | -- | -- | -- |
|  | | | | | | |
|  |  | **0:100** | **20:80** | **50:50** | **80:20** | **100:0** |
| 20 μM | **0:100** | -- | <0.001 | 0.002 | <0.001 | 0.005 |
|  | **20:80** | -- | -- | 0.206 | 0.020 | 0.042 |
|  | **50:50** | -- | -- | -- | <0.001 | 0.916 |
|  | **80:20** | -- | -- | -- | -- | <0.001 |
|  | **100:0** | -- | -- | -- | -- | -- |
|  | | | | | | |
|  |  | **0:100** | **20:80** | **50:50** | **80:20** | **100:0** |
| 100 μM | **0:100** | -- | 0.005 | 0.021 | <0.001 | 0.004 |
|  | **20:80** | -- | -- | 0.849 | 0.011 | 0.466 |
|  | **50:50** | -- | -- | -- | 0.043 | 0.762 |
|  | **80:20** | -- | -- | -- | -- | <0.001 |
|  | **100:0** | -- | -- | -- | -- | -- |
|  | | | | | | |
|  |  | **0:100** | **20:80** | **50:50** | **80:20** | **100:0** |
| 500 μM | **0:100** | -- | <0.001 | 0.009 | <0.001 | 0.004 |
|  | **20:80** | -- | -- | 0.463 | 0.033 | 0.141 |
|  | **50:50** | -- | -- | -- | 0.017 | 0.828 |
|  | **80:20** | -- | -- | -- | -- | <0.001 |
|  | **100:0** | -- | -- | -- | -- | -- |
|  | | | | | | |
|  |  | **0:100** | **20:80** | **50:50** | **80:20** | **100:0** |
| 1000 μM | **0:100** | -- | <0.001 | 0.004 | <0.001 | <0.001 |
|  | **20:80** | -- | -- | 0.809 | 0.104 | 0.296 |
|  | **50:50** | -- | -- | -- | 0.156 | 0.655 |
|  | **80:20** | -- | -- | -- | -- | 0.003 |
|  | **100:0** | -- | -- | -- | -- | -- |
|  | | | | | | |
|  |  | **0:100** | **20:80** | **50:50** | **80:20** | **100:0** |
| 2000 μM | **0:100** | -- | <0.001 | <0.001 | <0.001 | <0.001 |
|  | **20:80** | -- | -- | 0.660 | 0.157 | 0.478 |
|  | **50:50** | -- | -- | -- | 0.161 | 0.995 |
|  | **80:20** | -- | -- | -- | -- | 0.032 |
|  | **100:0** | -- | -- | -- | -- | -- |
