# Supplementary material for: The response of three-dimensional pancreatic alpha and beta cell co-cultures to oxidative stress
Source: PLoS One. 2022 Mar 15;17(3):e0257578. doi: 10.1371/journal.pone.0257578 (PMC8923503; doi:10.1371/journal.pone.0257578)
Supplement: S9 Table — (DOCX) [file pone.0257578.s009.docx]

**Table S9. Statistical significance (t-test) of the oxidative stress positive alphaTC1 cells in 3D aggregate co-cultures when exposed to 0–2000 μM H_2_O_2_.**

|  |  | **Ratio INS1E:alphaTC1** | | | | |
| --- | --- | --- | --- | --- | --- | --- |
|  |  | **0:100** | **20:80** | **50:50** | **80:20** | **100:0** |
| 0 μM | **0:100** | -- | 0.609 | 0.046 | 0.704 | <0.001 |
|  | **20:80** | -- | -- | 0.120 | 0.377 | <0.001 |
|  | **50:50** | -- | -- | -- | 0.038 | 0.006 |
|  | **80:20** | -- | -- | -- | -- | <0.001 |
|  | **100:0** | -- | -- | -- | -- | -- |
|  | | | | | | |
|  |  | **0:100** | **20:80** | **50:50** | **80:20** | **100:0** |
| 20 μM | **0:100** | -- | 0.469 | 0.270 | 0.264 | <0.001 |
|  | **20:80** | -- | -- | 0.557 | 0.038 | <0.001 |
|  | **50:50** | -- | -- | -- | 0.053 | 0.002 |
|  | **80:20** | -- | -- | -- | -- | <0.001 |
|  | **100:0** | -- | -- | -- | -- | -- |
|  | | | | | | |
|  |  | **0:100** | **20:80** | **50:50** | **80:20** | **100:0** |
| 100 μM | **0:100** | -- | 0.830 | 0.918 | 0.036 | 0.002 |
|  | **20:80** | -- | -- | 0.801 | 0.013 | 0.004 |
|  | **50:50** | -- | -- | -- | 0.186 | 0.004 |
|  | **80:20** | -- | -- | -- | -- | <0.001 |
|  | **100:0** | -- | -- | -- | -- | -- |
|  | | | | | | |
|  |  | **0:100** | **20:80** | **50:50** | **80:20** | **100:0** |
| 500 μM | **0:100** | -- | 0.075 | 0.338 | 0.658 | <0.001 |
|  | **20:80** | -- | -- | 0.827 | 0.007 | <0.001 |
|  | **50:50** | -- | -- | -- | 0.134 | 0.001 |
|  | **80:20** | -- | -- | -- | -- | <0.001 |
|  | **100:0** | -- | -- | -- | -- | -- |
|  | | | | | | |
|  |  | **0:100** | **20:80** | **50:50** | **80:20** | **100:0** |
| 1000 μM | **0:100** | -- | 0.407 | 0.659 | 0.724 | <0.001 |
|  | **20:80** | -- | -- | 0.927 | 0.133 | <0.001 |
|  | **50:50** | -- | -- | -- | 0.385 | 0.002 |
|  | **80:20** | -- | -- | -- | -- | <0.001 |
|  | **100:0** | -- | -- | -- | -- | -- |
|  | | | | | | |
|  |  | **0:100** | **20:80** | **50:50** | **80:20** | **100:0** |
| 2000 μM | **0:100** | -- | 0.822 | 0.715 | 0.363 | 0.001 |
|  | **20:80** | -- | -- | 0.731 | 0.390 | <0.001 |
|  | **50:50** | -- | -- | -- | 0.862 | <0.001 |
|  | **80:20** | -- | -- | -- | -- | <0.001 |
|  | **100:0** | -- | -- | -- | -- | -- |
