# Supplementary material for: The response of three-dimensional pancreatic alpha and beta cell co-cultures to oxidative stress
Source: PLoS One. 2022 Mar 15;17(3):e0257578. doi: 10.1371/journal.pone.0257578 (PMC8923503; doi:10.1371/journal.pone.0257578)
Supplement: S10 Table — (DOCX) [file pone.0257578.s010.docx]

**Table S10. Statistical significance (t-test) of the intracellular GSH levels in 3D aggregate co-cultures when exposed to 500 μM H_2_O_2_ (Fig 5).**

|  |  | **0 μM H_2_O_2_** | | | **500 μM H_2_O_2_** | | |
| --- | --- | --- | --- | --- | --- | --- | --- |
|  | **INS1E:alphaTC1** | **0:100** | **50:50** | **100:0** | **0:100** | **50:50** | **100:0** |
| **0 μM H_2_O_2_** | **0:100** | -- | 0.199 | 0.048 | 0.016 | -- | -- |
|  | **50:50** | -- | -- | 0.258 | -- | 0.216 | -- |
|  | **100:0** | -- | -- | -- | -- | -- | 0.121 |
| **500 μM H_2_O_2_** | **0:100** | -- | -- | -- | -- | 0.534 | 0.626 |
|  | **50:50** | -- | -- | -- | -- | -- | 0.951 |
|  | **100:0** | -- | -- | -- | -- | -- | -- |
| **0 μM H_2_O_2_** | **0:100** | -- | 0.207 | 0.338 | 0.234 | -- | -- |
|  | **50:50** | -- | -- | 0.642 | -- | 0.070 | -- |
|  | **100:0** | -- | -- | -- | -- | -- | 0.100 |
| **500 μM H_2_O_2_** | **0:100** | -- | -- | -- | -- | 0.599 | 0.581 |
|  | **50:50** | -- | -- | -- | -- | -- | 0.981 |
|  | **100:0** | -- | -- | -- | -- | -- | -- |
