# Supplementary figures and images for: The response of three-dimensional pancreatic alpha and beta cell co-cultures to oxidative stress
Source: PLoS One. 2022 Mar 15;17(3):e0257578. doi: 10.1371/journal.pone.0257578 (PMC8923503; doi:10.1371/journal.pone.0257578)

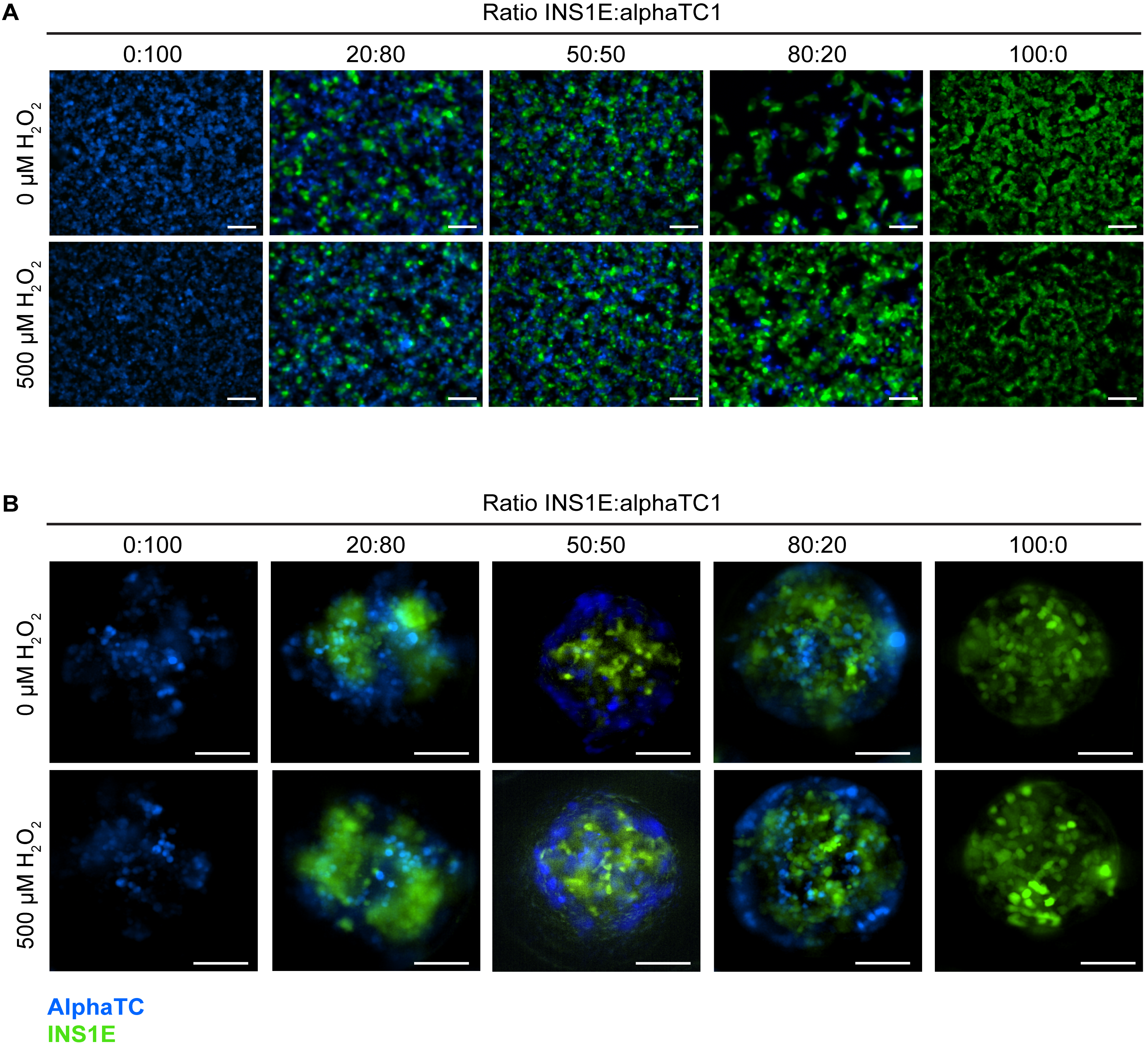

Supplement: S1 Fig — A) H2O2 did not affect the shrinking and swelling of the INS1E and alphaTC1 cells in the different ratios in a monolayer. B) H2O2 also did not affect the shrinking and swelling of the INS1E and alphaTC1 cells in the different ratios of the 3D aggregates. N = 3 and a typical example is shown. (TIF) [file pone.0257578.s011.tif]

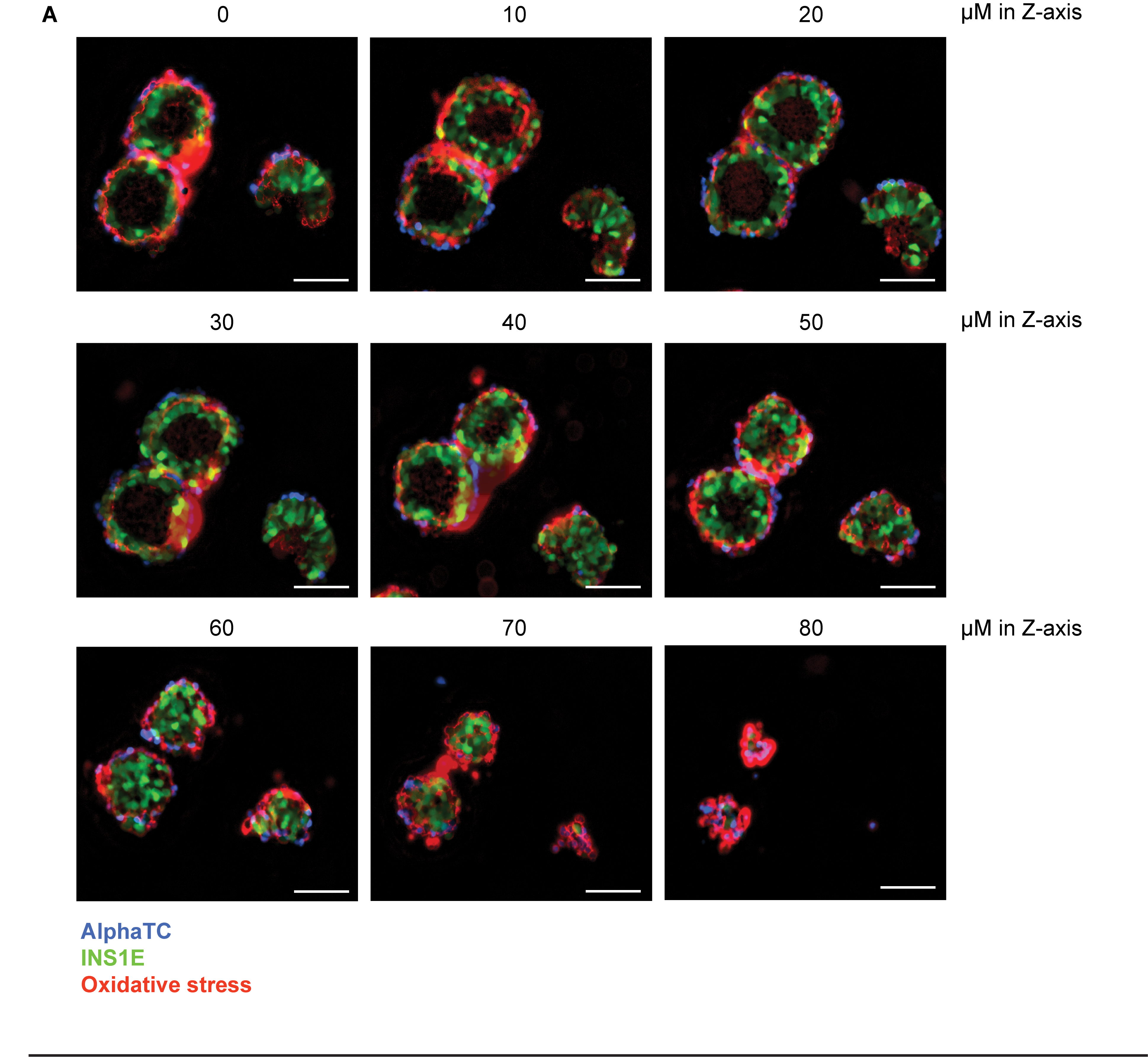

Supplement: S2 Fig — A) When exposed to 1000 μM H2O2, independent of the Z-stack number, cells within the 3D aggregate were coloured red because of the oxidative stress induced. A typical example is shown. (TIF) [file pone.0257578.s012.tif]

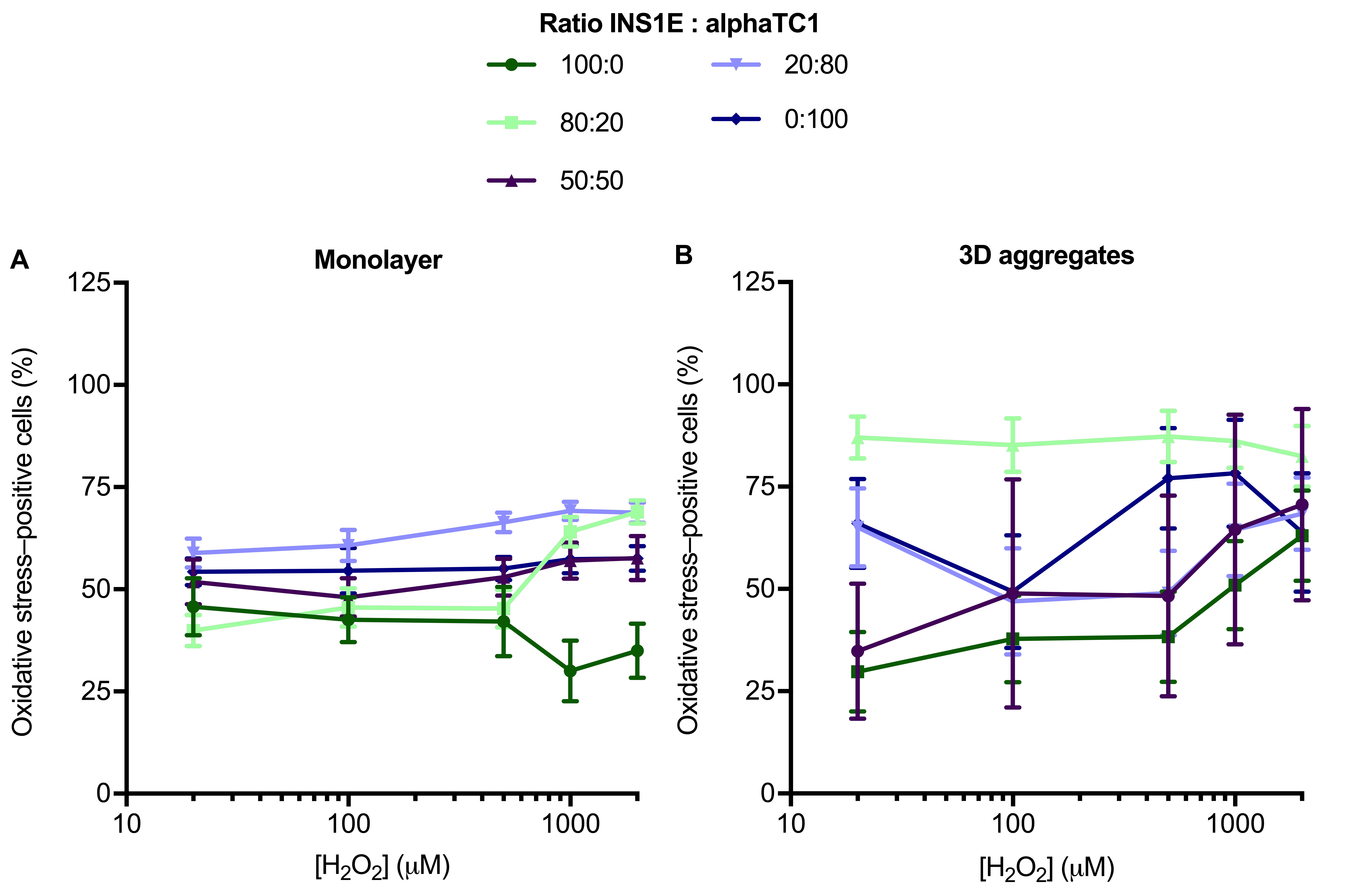

Supplement: S3 Fig — A) In a monolayer, oxidative stress induced by H2O2 increased the percentage of oxidative stress–positive cells in the ratios of 80:20 and 20:80 INS1E:alphaTC1. B) In 3D aggregates, oxidative stress induced by H2O2 did not increase the total percentage of oxidative stress–positive cells in the ratios 100:0, 80:20, 50:50, 20:80 and 0:100 INS1E:alphaTC1. N = 3, A) n≥1; B) n≥2 and data are presented as mean ± SEM. All p-values are shown in S3 and S4 Tables. (TIFF) [file pone.0257578.s013.tiff]
